# Transcriptome profile of liver at different physiological stages reveals potential mode for lipid metabolism in laying hens

**DOI:** 10.1186/s12864-015-1943-0

**Published:** 2015-10-09

**Authors:** Hong Li, Taian Wang, Chunlin Xu, Dandan Wang, Junxiao Ren, Yanmin Li, Yadong Tian, Yanbin Wang, Yuping Jiao, Xiangtao Kang, Xiaojun Liu

**Affiliations:** College of Animal Science and Veterinary Medicine, Henan Agricultural University, Zhengzhou, 450002 China; Henan Innovative Engineering Research Center of Poultry Germplasm Resource, Zhengzhou, 450002 China; International Joint Research Laboratory for Poultry Breeding of Henan, Henan Agricultural University, Zhengzhou, 450002 China; Institute of Animal Husbandry and Veterinary Medicine, Henan Academy of Agricultural Sciences, Zhengzhou, 450002 China

**Keywords:** RNA-Seq, Laying hens, Liver, Fat metabolism, Function analysis

## Abstract

**Background:**

Liver is an important metabolic organ that plays a critical role in lipid synthesis, degradation, and transport; however, the molecular regulatory mechanisms of lipid metabolism remain unclear in chicken. In this study, RNA-Seq technology was used to investigate differences in expression profiles of hepatic lipid metabolism-related genes and associated pathways between juvenile and laying hens. The study aimed to broaden the understanding of liver lipid metabolism in chicken, and thereby to help improve laying performance in the poultry industry.

**Results:**

RNA-Seq analysis was carried out on total RNA harvested from the liver of juvenile (*n* = 3) and laying (*n* = 3) hens. Compared with juvenile hens, 2567 differentially expressed genes (1082 up-regulated and 1485 down-regulated) with *P* ≤ 0.05 were obtained in laying hens, and 960 of these genes were significantly differentially expressed (SDE) at a false discovery rate (FDR) of ≤0.05 and fold-change ≥2 or ≤0.5. In addition, most of the 198 SDE novel genes (91 up-regulated and 107 down-regulated) were discovered highly expressed, and 332 SDE isoforms were identified. Gene ontology (GO) enrichment and KEGG (Kyoto Encyclopedia of Genes and Genomes) pathway analysis showed that the SDE genes were most enrichment in steroid biosynthesis, PPAR signaling pathway, biosynthesis of unsaturated fatty acids, glycerophospholipid metabolism, three amino acid pathways, and pyruvate metabolism (*P* ≤ 0.05). The top significantly enriched GO terms among the SDE genes included lipid biosynthesis, cholesterol and sterol metabolic, and oxidation reduction, indicating that principal lipogenesis occurred in the liver of laying hens.

**Conclusions:**

This study suggests that the majority of changes at the transcriptome level in laying hen liver were closely related to fat metabolism. Some of the SDE uncharacterized novel genes and alternative splicing isoforms that were detected might also take part in lipid metabolism, although this needs further investigation. This study provides valuable information about the expression profiles of mRNAs from chicken liver, and in-depth functional investigations of these mRNAs could provide new insights into the molecular networks of lipid metabolism in chicken liver.

**Electronic supplementary material:**

The online version of this article (doi:10.1186/s12864-015-1943-0) contains supplementary material, which is available to authorized users.

## Background

Liver is the main metabolic organ where more than 70 % of the *de novo* synthesis of fatty acids occurs in chicken [1–3]. Especially, the liver plays an important role in lipid synthesis, degradation, and transport processes. During the hen laying cycle, hydrophobic lipids including triacylglycerols, cholesteryl esters, cholestery esters, and free fatty acids are synthesized in the liver and assembled to form egg-yolk precursors such as very-low density lipoprotein (VLDL) and vitellogenin particles. The particles are then secreted into the circulation and transferred to the developing oocyte to meet the requirements for embryo growth and development [4–7]. The molecular regulatory mechanisms of these crucial physiological processes have been investigated extensively since the 1970s, and thus are reasonably well understood [8–11].

It is generally agreed that the physiological processes for lipids synthesis, secretion, and transfer in the liver of laying hens are regulated tightly by estrogen. Estrogen regulates the transcription of target genes containing consensus estrogen response elements through the estrogen receptors (ERs) ERα, ERβ, and G protein-coupled receptor (GPR30) [12–14]. The different receptors play distinct roles in gene regulation [13]. Previous studies have revealed that estrogen physiological functions could be mediated by different receptors in various species [15, 16]. However, the specific nuclear receptor subtype that mediates the production of yolk precursors in chicken liver is unclear [17].

Although most of the genes and their products involved in hepatic lipid metabolism are highly similar in poultry and mammalian species, the functions of some of these genes and their products are considered to be slightly different in poultry compared with their functions in mammals [4, 18–20]. For instance, a recent study on lysophosphatidylglycerol acyltransferase 1 (LPGAT1) indicated that LPGAT1 may play an important role in lipid synthesis in mice [21] rather than in poultry. Moreover, it has been suggested that poultry species may have lost some of the genes related to lipid metabolism during the evolutionary process [22]. Therefore, the range of genes and their products involved in hepatic lipid metabolism in laying hen remains to be fully elucidated [23].

How VLDL particles are assembled and secreted in chicken liver is still not fully understood. In mammals, it has been well documented that microsomal triglyceride transfer protein (MTTP) assists in lipoprotein assembly to form low-density lipoprotein [21, 24–28]. The formation of VLDL particles in avian species is tightly regulated by estrogen, and a previous study demonstrated that the up-regulation of *MTTP* in liver was not required for increased VLDL assembly during the laying period in chicken [29]. Therefore, understanding the synthesis, formation, and transport of yolk precursors in the liver of laying hens is important.

In recent years, the study of gene regulation and interactions has broadened considerably because of advances in genomics, epigenomics, and bioinformatics, as well as with the development of next generation sequencing. RNA-Seq is a novel gene expression profiling technology based on high-throughput sequencing [30]. Compared with other large-scale gene expression profiling methods, RNA-Seq is superior in detecting mRNA expression in different tissues or at different development stages in a single assay, which can help reveal novel genes and splice variants [31].

In this study, expression profiles of hepatic lipid metabolism-related genes and associated pathways were investigated between juvenile and laying hens (two different physiological stages) using RNA-Seq technology. Because lipogenesis is known to be highly stimulated in the liver of sexually mature hens and to eliminate genes that may be unrelated to lipid metabolism, liver expression profiles were compared between juvenile hens and laying hens. Bioinformatics tools were used to analyze the major differentially expressed genes and pathways. The present study provides an overview of the genes related to lipid metabolism that play a significant role during embryonic development by synthesizing components of the egg yolk.

## Methods

### Animals and liver tissue samples preparation

All animal experiments were performed in accordance with the protocol approved by the Institutional Animal Care and Use Committee (IACUC) of Henan Agricultural University. The experimental animals used in this study were one strain of the Chinese domestic breed laying hens (Lushi green shell chicken).

All the chickens were raised in cages under the same environment with *ad libitum* conditions. Six hens were selected randomly from two different physiological stages, juvenile hens and laying hens. The three juvenile hens were slaughtered when they were 20 weeks old (L20), and the three laying hens were slaughtered when they were 30 weeks old (L30). Liver tissue samples were harvested immediately. The collected samples were immediately snap-frozen in liquid nitrogen and stored at −80 °C for further use.

### RNA extraction

Total RNA was extracted from the chicken liver tissues using TRIzol® reagents following the manufacturer’s manual (Invitrogen, Carlsbad, CA). Degradation and contamination of the total RNA was detected on 1 % agarose gels. The purity of the total RNA was assessed using a NanoPhotometer® spectrophotometer (IMPLEN, CA). The integrity was estimated using a RNA Nano 6000 Assay Kit with the Agilent Bioanalyzer 2100 system (Agilent technologies, Santa Clara, CA). The RNA concentration was checked with a Qubit® RNA Assay Kit in a Qubit® 2.0 flurometer (Life Technologies, CA). The 28S/18S ratio of the qualified RNA ranged from 1.8 to 2.0 and the RNA integrity values ranged from 8.0 to 10.0. RNA samples were stored at −80 °C for further analysis.

### RNA-Seq library construction and sequencing

Six mRNA libraries were constructed, one for each of the samples (L20-1, L20-2, L20-3 and L30-1, L30-2, L30-3). A total of 3 μg RNA per sample was prepared for mRNA sequencing using the TruSeq RNA Sample Prep Kit v2 (Illumina) according to the manufacturer’s protocol. Briefly, the mRNA was isolated from the total RNA using oligo (dT) beads with two rounds of oligo-dT purification. Following the rRNA depletion step, the purified RNA was fragmented with the Ribo-Zero rRNA Removal Kits (Epicentre). First-strand cDNA synthesis was performed using the Invitrogen random hexamer primers and Superscript II reverse transcriptase (Invitrogen). The second-strand was synthesized using Invitrogen DNA polymerase 1 (Invitrogen). End repair and poly-adenylation were performed, and the mRNAs were ligated to adapters before PCR amplification. The enriched cDNA templates that were 100 nucleotides (nt) long were purified and used for further analysis. The libraries were qualified using a Qubit® 2.0 Fluorometer (Invitrogen) and Qubit dsDNA HS Assay Kit (Invitrogen). The purity and size of the libraries were checked on an Agilent 2100 Bioanalyzer (Agilent Technologies). The adapter-ligated cDNA fragment libraries were run on an Illumina GAIIx analyzer to complete the cluster generation and primer hybridization. Then the Illumina PE flow cell (v3-HS) carrying clusters were sequenced with paired-end 2 × 100 nt multiplex on an Illumina HiSeq 2500 platform following the manufacturer’s instruction (Illumina).

### Transcriptome sequencing data processing and annotation

After the sequencing was completed, image data was outputted and transformed into raw reads and stored with a FASTQ format. The obtained raw reads were cleaned using the FASTX-Toolkit (version: 0.0.13) [32]. Reads with adapter, low quality at 3′ end, containing fuzzy N bases, rRNA, sequences shorter than 20 nt and low quality with Q <20 were removed. The resultant clean reads from each sample library were used for the downstream analyses. The clean reads were mapped to the chicken genome assembly (galGal4), which we downloaded from Ensembl [33], using the spliced mapping algorithm in TopHat2 (version: 2.0.9) [34]. We used TopHat2 as the mapping tool because it can generate a database of splice junctions based on Ensemble annotations of galGal4 and thus can produce a better mapping result than other non-splice mapping tools.

### Transcript identification and alternative splicing analysis

We used the reference annotation-based transcript (RABT) assembly method in Cufflink (version: 2.1.1) [35] to construct and identify both known and novel transcripts from the TopHat2 alignment results. The AStalavist software (version: 3.2) [36, 37] can characterize alternative splicing (AS) for whole transcriptome data from reference annotated transcripts. We used AStalavist to estimate AS events within and between groups. The differentially expressed isoforms were estimated by Cufflink.

### Quantification of differential mRNA expression levels

The expression levels of the mapped genes were estimated from the transcriptome sequencing data based on the number of raw reads. HTSeq (version: 0.6.1) [38] was used to count the numbers of reads mapped to each gene. The reads for each gene were normalized by using fragments per kilo base of exon model per million mapped reads (FPKM). The quantification and differential analyses were conducted according to the Cufflink (version: 2.1.1) program. The criteria normalization formula is as follows:

$$ \mathrm{FPKM}=\frac{\mathrm{transcription}\ \mathrm{reads}}{\mathrm{transcription}\ \mathrm{length}}\times \mathrm{total}\ \mathrm{mapped}\ \mathrm{reads}\ \mathrm{in}\ \mathrm{run}\times {10}^9 $$

The Cuffdiff was used to analyze the differential expression genes. In our study, the false discovery rate (FDR) was used to determine the threshold of the *P*-value in multiple tests and analyses. Genes were identified as differentially expressed (DE) genes when *P* ≤ 0.05. DE genes with fold changes ≥2 or ≤0.5 (FDR ≤0.05) were identified as significantly differentially expressed (SDE) genes [39].

### Quantitative real time PCR (qRT-PCR)

To confirm the repeatability and accuracy of the RNA-Seq gene expression data obtained from the chicken liver libraries, qRT-PCR was carried out on 12 randomly selected DE genes that were prepared from the total RNA. The PrimeScript™ RT Reagent kit with gDNA Eraser (TaKaRa, Dalian, China) was used to synthesize the first-strand cDNA. The qRT-PCRs were performed on a LightCycler® 96 Real-Time PCR system (Roche Applied Science) in a 20-μl reaction volume containing 2 μL cDNA, 10 μL 2 × SYBR®Premix Ex Taq™ II (TliRNaseH Plus) (TaKaRa), 0.5 μL each of forward and reverse primers (10 μM), and 7 μL deionized water. The *β-actin* gene was used as the reference gene, and all the qRT-PCR gene-specific primers were designed using the Oligo 6.0 software [40]. The primer sequences are presented in Additional file 1: Table S1. The qPCR amplification procedure was as follows: 95 °C for 3 min, 40 cycles of 95 °C for 12 s, 61 °C for 40 s, 72 °C for 30 s, and an extension for 10 min at 72 °C. All the reactions were run with three replicates, and the relative gene expression levels were analyzed using the comparative *C*_T_ method (also referred to as the 2^-△△*C*T^ method) [41]. In this study, the Wilcox rank sum test was used. The statistical analyses were performed with R for windows version 3.2.0 [42], with the test conducted as a one-sided tail test and a significance level of *P* ≤ 0.05. The values are presented as mean ± standard error.

### Functional annotation analyses

Functional enrichment of the SDE genes was analyzed using the web-based tools in DAVID [43] to identify enriched gene ontology (GO) terms and Kyoto Encyclopedia of Genes and Genomes (KEGG) pathways, group functionally related genes, and cluster the annotation terms with a retained of EASE scores 0.1 [44, 45]. The *P-*value was calculated as

$$ P=1-{\displaystyle {\sum}_{i=0}^{m-1}}\frac{\left(\begin{array}{c}\hfill M\hfill \\ {}\hfill i\hfill \end{array}\right)\left(\begin{array}{c}\hfill N-M\hfill \\ {}\hfill n-i\hfill \end{array}\right)}{\left(\begin{array}{c}\hfill N\hfill \\ {}\hfill n\hfill \end{array}\right)} $$

where *N* is the total number of genes in the genome, *n* is the total number of SDE genes, *M* is the number of genes annotated with a certain GO term, and *m* is the number of SDE genes annotated with the same certain GO term. Only the GO terms and KEGG pathways with *P* ≤ 0.05 were taken into account as significantly enriched among the SDE genes [46].

## Results

### Identification of expressed transcripts in the chicken liver transcriptome

**Fig. 1:**
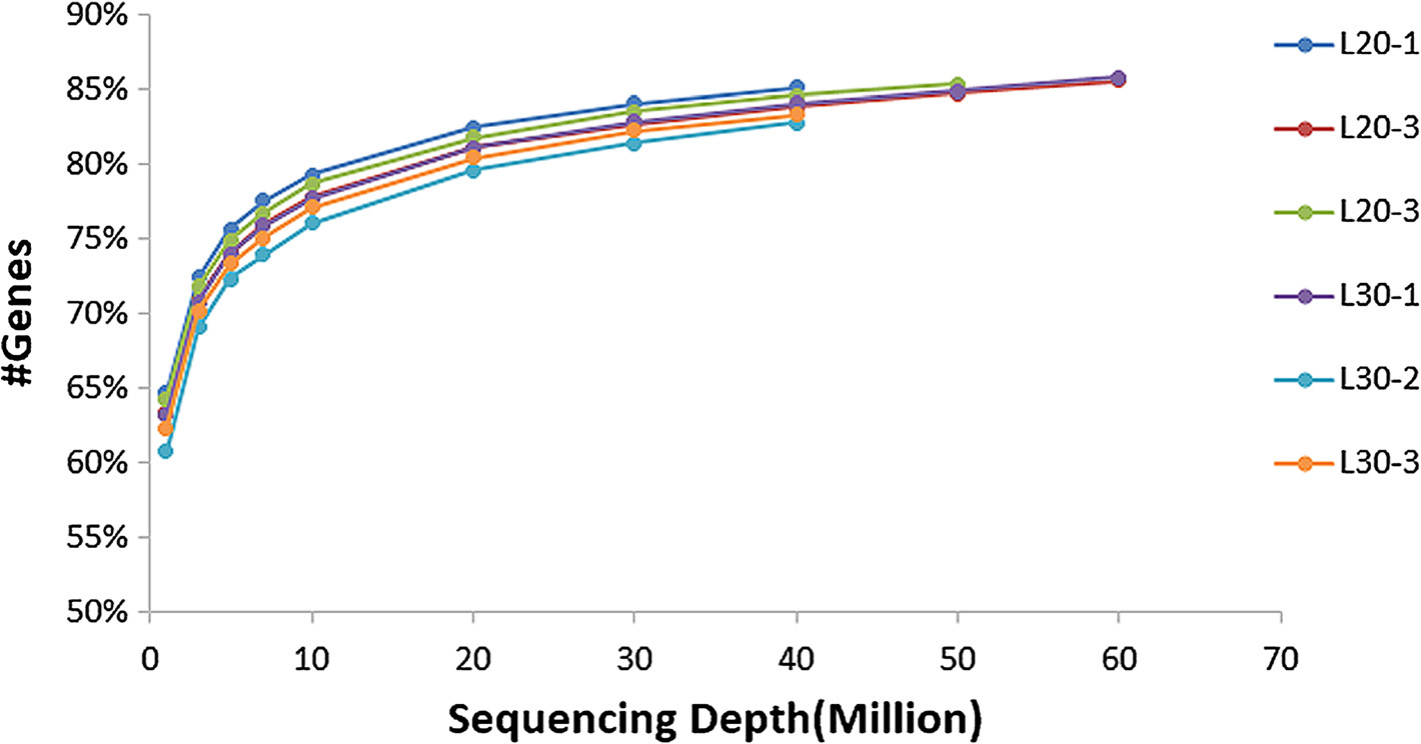
Saturation analysis of the transcriptome sequencing data from six chicken liver libraries. L20-1, L20-2, and L20-3 from the liver of 20 week-old juvenile hens and L30-1, L30-2, and L30-3 from 30 week-old laying hens. x-axis, sequencing depth; y-axis, proportion of covered genes

**Fig. 2:**
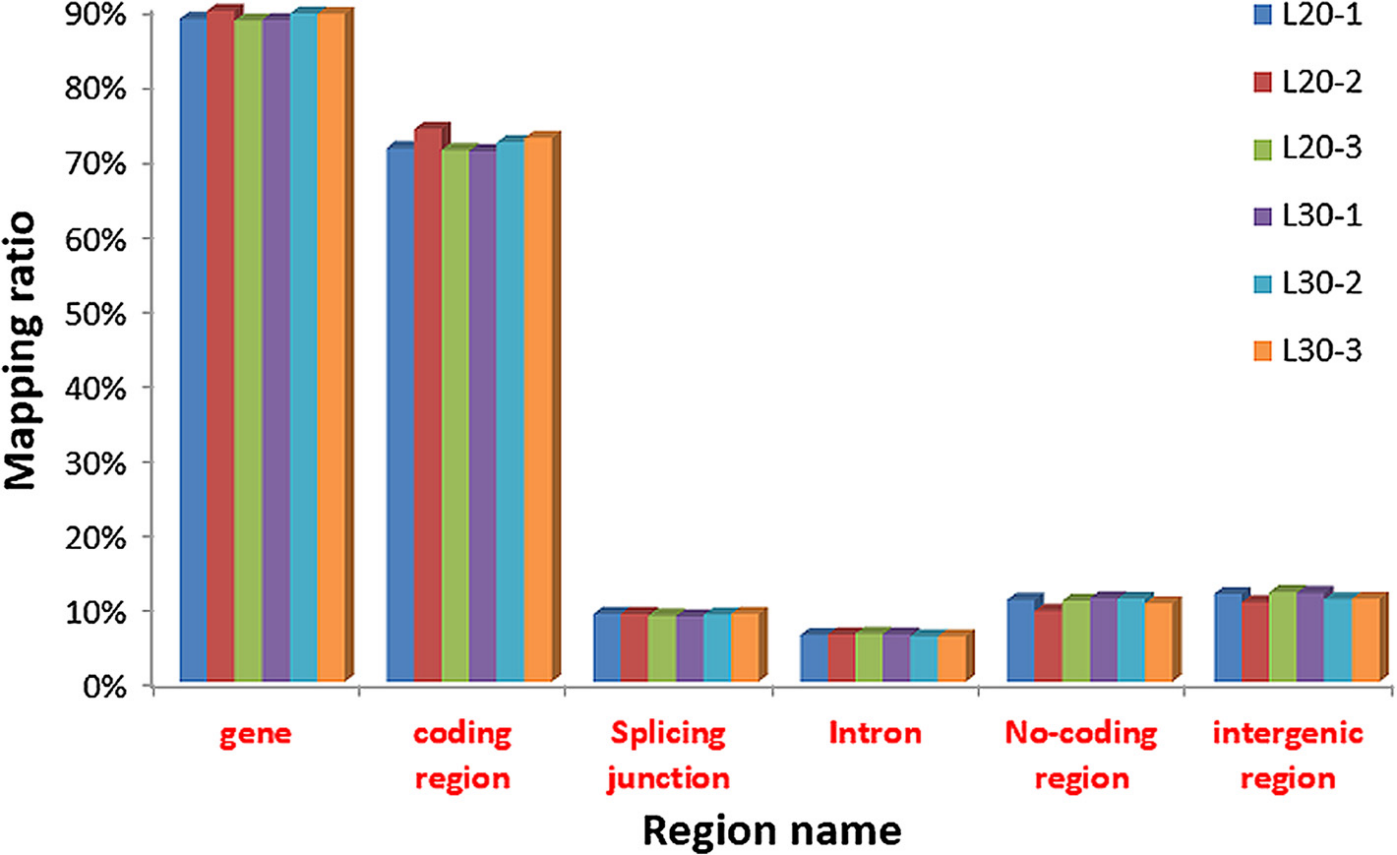
Distribution of the mapped reads on different regions of the chicken reference genome. Non-coding regions include all the 5′UTR, 3′UTR and other non-coding RNA regions

In this study, we established six cDNA libraries L20-1, L20-2, and L20-3 from the liver of 20-week-old juvenile hens and L30-1, L30-2, and L30-3 from 30-week-old laying hens that represented two different physiological stages. The RNA-Seq generated from 42,113,152 to 67,296,120 raw reads for each library, with an average of 54,373,054 and 50,986,088 paired-end reads for the L20 and L30 groups, respectively. The sequencing depth of 40 M reads for each library was saturated (Fig. 1). After filtering the low quality reads, the average numbers of clean reads were 51,554,387 (94.8 %) and 48,351,463 (94.8 %) for the L20 and L30 groups, respectively. The clean reads were used for all further analyses. After assembly, 13,523 mRNAs were obtained from the two groups; 13,519 (99.97 %) were found in the juvenile hen libraries and 13,436 (99.36 %) were found in the laying hen libraries and 13,432 of these mRNAs were commonly expressed between the two groups. Approximately 85 % of the reads in each library were uniquely mapped to the galGal4 assembly of the chicken genome, and the average mapping rates were 83.0 and 84.2 % for the L20 and L30 groups, respectively (Table 1). The density of the mapped reads on different regions of the genome is displayed in Fig. 2.

**Table 1:** Characteristics of the reads from six chicken liver libraries

Sample ID^a^	Raw bases (Gb)	Q20 value (%)	GC content (%)	Raw reads	Clean reads	Mapped reads	Mapped unique reads^b^	Mapping ratio (%)^c^
L20–1	4.5	95.4	47	44674654	42381020	36083033	35118821	85.1
L20–2	6.5	95.3	47	65393800	61933858	52824621	51312296	85.3
L20–3	5.3	95.5	47	53050708	50348282	43068094	41902030	85.5
L30–1	6.7	95.5	46	67296120	63775556	54827754	53528180	86.0
L30–2	4.2	95.5	46	42113152	39958736	34571635	33690669	86.5
L30–3	4.4	95.6	46	43548992	41320096	35784180	34935615	86.6

^a^L20, liver samples from juveniles; L30, liver samples from egg laying hens. ^b^Mapped unique reads, reads that matched the reference genome in only one position. ^c^Mapping ratio, mapped reads/all reads

**Fig. 3:**
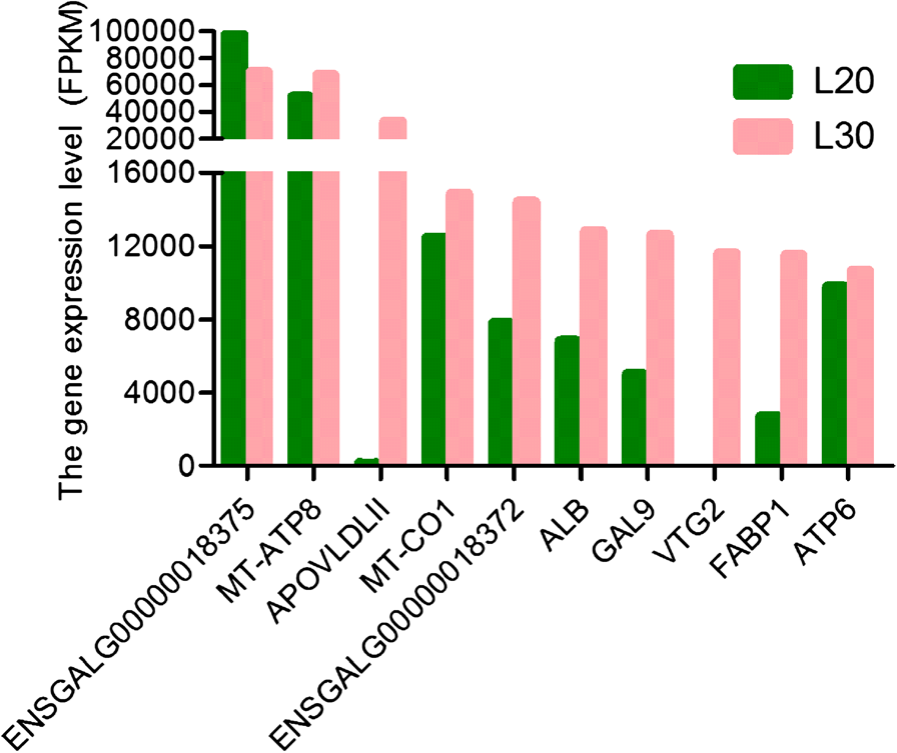
Top ten most abundantly expressed mRNAs in liver libraries from juvenile and laying hens. FPKM values of up to 10,000 genes are shown

The top ten most abundantly expressed genes in both groups (FPKM from 10,643 to 69,528 reads) ranked by absolute abundance were ENSGALG00000018375 (uncharacterized protein), ATP synthase protein 8 (*ATP8*), apovitellenin 1 (*APOVLDLII*), cytochrome c oxidase subunit 1 (*COX1*), ENSGALG00000018372 (uncharacterized protein), serum albumin (*ALB*), gallinacin-9 (*GAL9*), vitellogenin 2 (*VTG2*), fatty acid binding protein 1 (*FABP1*), and *ATP6*. The expression levels of *APOVLDLII* and *VTG2* were much lower in the liver of juvenile hens than in laying hens (Fig. 3).

The correlation of transcript expression levels between samples is a crucial indicator for the reliability of the experimental results and the rationality of sampling. Generally, the Pearson correlation coefficient shall be no less than 0.92 (r^2^ ≥ 0.92) [47]. We performed correlation analyses among the six samples to determine whether differential gene expression was observed between the L20 and L30 groups. The Pearson correlation coefficient demonstrated that the expression levels of the three biological replicates in each group (L20 and L30) were similar based on the normalized FPKM values (i.e., all r^2^ ≥ 0.93).

### Identification of differentially expressed genes and isoforms between the two physiological development stages

In this study, we identified a total of 13,532 genes in the chicken liver libraries; 1767 of them were novel genes and 198 of these novel genes showed significant changes in expression (91 up-regulated and 107 down-regulated) between the L20 and L30 groups (FDR ≤0.05) (Additional file 2: Table S2). Among the annotated genes, we identified 2567 DE genes (1082 up-regulated and 1485 down-regulated) in L30 compared with L20 with *P* ≤ 0.05; 960 of these were SDE genes (473 up-regulated and 487 down-regulated) with a fold-change ≥2 or ≤0.5 (FDR ≤0.05) (Additional file 3: Table S3).

**Fig. 4:**
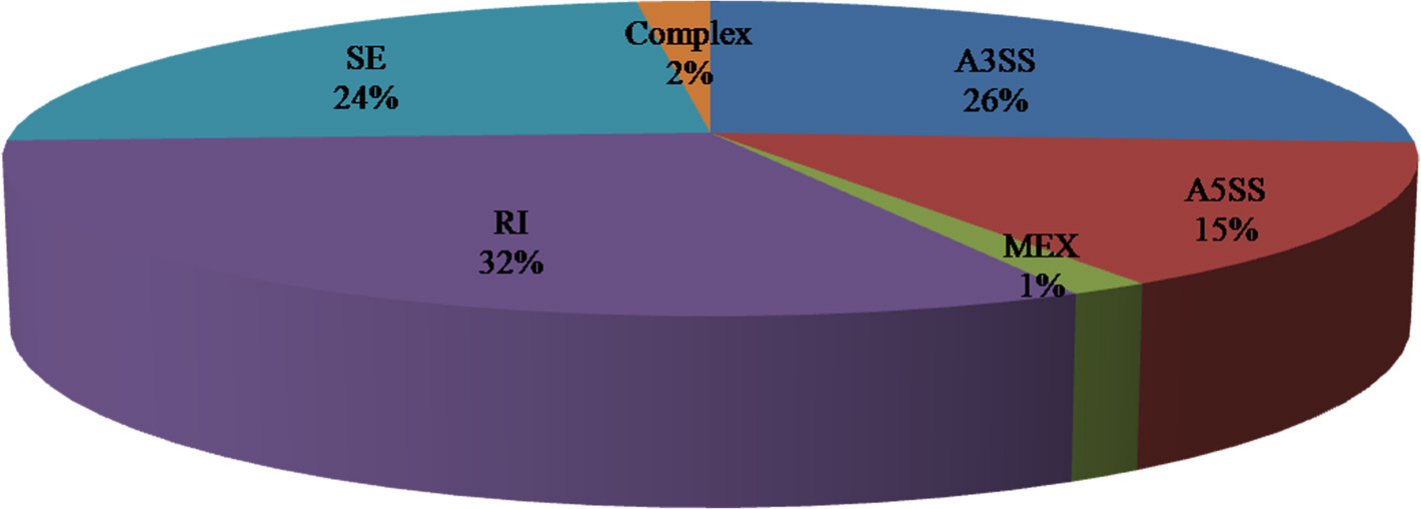
Distribution of alternative splicing isoforms in liver libraries from juvenile and laying hens

In mammals, splice variants are considered to be primary drivers of the evolution of phenotypic complexity [48–50]. We detected a total 14,212 splice variants in both groups. A total of 332 DE isoforms (115 down-regulated and 217 up-regulated; FDR ≤0.05) were detected in L30 compared with L20, and 287 (86.4 %) of them were annotated (Additional file 4: Table S4). The chromosomal position of each transcript was obtained by aligning the sequence to the chicken reference genome. The analysis detected six different splice patterns in the chicken liver transcriptome data, namely skipped exon (SE), alternative 5′ splicing site (A5SS), alternative 3′ splicing site (A3SS), retained intron (RI), mutually exclusive exon (MEX), and complex. Four of these splice patterns, skipped exon, alternative 5′ and 3′splicing sites, and retained intron were the major splicing patterns found in our study, representing 96.5 % of the total AS events; mutually exclusive exon and complex were rare events and accounted for only 3.5 % of the AS events (Fig. 4). The average number of alternative transcripts per chromosome was 721, and chromosomes 16 (93 transcripts) and W (eight transcripts) had the smallest numbers of alternative transcripts.

### Real-time PCR validation of differential genes expression

To confirm the accuracy of the RNA-Seq transcriptome data, 12 genes were selected randomly including four significantly up-regulated genes, three significantly down-regulated genes, and five genes with no significant differential expression. The expression levels of the selected genes were quantified using qRT-PCR, and the results were consistent with the findings obtained by RNA-Seq (Table 2). The results suggest that the RNA-Seq reliably identified DE mRNAs and revealed novel genes in the chicken liver transcriptome.

**Table 2:** Expression patterns of the 12 mRNAs selected for qRT-PCR validation

Gene name	qRT-PCR		RNA-Seq	
	Fold-change^a^ (L30/L20^b^)	*P*-value	Fold-change (L30/L20)	*P*-value
*APAO1*	−0.48	0.008	−0.34	0.004
*CEPT1*	3.71	0.004	2.73	0.000
*CETP*	−0.05	0.010	−0.06	0.000
*PRDX*	0.61	0.056	0.94	0.732
*RPL6*	0.71	0.281	1.31	0.177
*RPS24*	0.97	0.192	1.27	0.177
*FOXO3*	2.43	0.006	3.86	0.000
*SIRT1*	2.62	0.054	1.32	0.087
*MTP*	1.51	0.068	1.12	0.537
*ApoB*	14.91	0.006	8.03	0.000
*LPGAT1*	−0.47	0.050	−0.64	0.010
*ENSGALG-*	159.23	0.000	92.77	0.000
*00000010018*				

^a^Minus sign indicates the gene was down-regulated. ^b^L30/L20, fold change in gene expression in liver samples from egg laying hens (L30) compared with liver samples from juveniles (L20)

### Functional analysis of differentially expressed genes

To better understand the regulation network of lipid synthesis and transport during egg production, we analyzed the functional distribution of the DE genes in the liver of laying hens liver compared with the liver of juvenile hens.

**Fig. 5:**
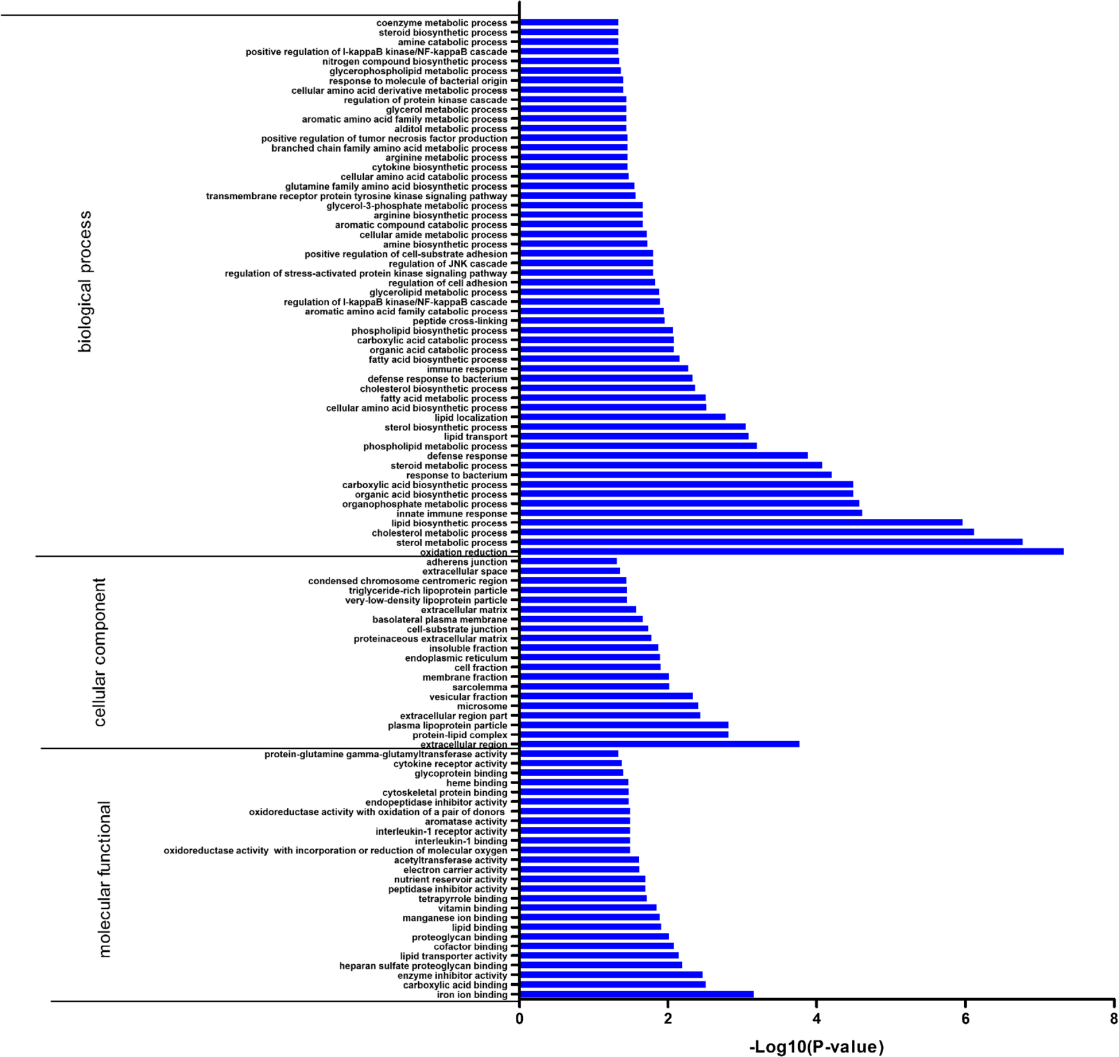
GO enrichment analysis of SDE genes in the liver transcriptome of laying hens. Only the significantly enriched (*P* ≤ 0.05) biological process, cellular component, and molecular function categories are shown

We detected 960 SDE genes in L30 compared with L20 and clustered them based on the GO and KEGG pathway analyses. The percentages of the SDE genes involved in the GO biological process, molecular function, and cellular component categories were 46.4, 50.7, and 30.7 %, respectively. We obtained a total of 113 clusters based on the GO functional annotation of the SDE genes (Additional file 5: Table S5). The cluster with the highest score was most enrichment in sterol, cholesterol, and steroid metabolic and biosynthetic processes, lipid metabolism, lipid localization, protein-lipid complex, plasma lipoprotein particle, VLDL particle, and triglyceride-rich lipoprotein particle (Table 3). Thus, the GO term enrichment analysis showed that the SDE genes were significantly enriched in oxidation reduction, sterol and cholesterol metabolic processes, and lipid biosynthetic processes (Fig. 5). SDE genes enriched in terms related to fat metabolism included apolipoprotein B (*ApoB*), apolipoprotein A-I (*APOA1*), lecithin-cholesterol acyltransferase (*LCAT*), insulin induced gene 1 (*INSIG1*), and *VLDLR*. SDE genes enriched in terms related to signal, disulfide bond, secreted and storage protein included *VLDL*, *VTG1*, *VTG2*, and *APOVLDII*.

**Table 3:** Top gene ontology clusters of SDE genes between liver samples from juvenile and laying hens

Category^a^	Term ID	Term	Genes	*P*-value
GOTERM_BP_FAT	GO:0016125	Sterol metabolic process	*APOB, APOA1, HMGCR, CYP7A1, LCAT, INSIG1, FDPS, LSS, SC4MOL, VLDLR, DHCR24*	1.68E-07
GOTERM_BP_FAT	GO:0008203	Cholesterol metabolic process	*APOB, APOA1, HMGCR, CYP7A1, LCAT, INSIG1, FDPS, LSS, VLDLR, DHCR24*	7.58E-07
SP_PIR_KEYWORDS		Lipid metabolism	*FAR1, APOB, APOA1, LCAT, INSIG1, ACSBG2, AACS, VLDLR*	1.15E-05
GOTERM_BP_FAT	GO:0008202	Steroid metabolic process	*OSBPL3, HMGCR, FDPS, LSS, SC4MOL, APOB, APOA1, LCAT, CYP7A1, INSIG1, OSBPL10, VLDLR, DHCR24*	8.40E-05
SP_PIR_KEYWORDS		Cholesterol metabolism	*APOB, APOA1, LCAT, INSIG1, VLDLR*	7.97E-04
GOTERM_BP_FAT	GO:0006869	Lipid transport	*APOB, TPRXL, APOA1, PPARG, ANXA1, ATP11A, CETP, VTG2, ATP8B3, VLDLR*	8.29E-04
GOTERM_BP_FAT	GO:0016126	Sterol biosynthetic process	*HMGCR, FDPS, LSS, SC4MOL, DHCR24*	8.92E-04
SP_PIR_KEYWORDS		Steroid metabolism	*APOB, APOA1, LCAT, INSIG1, VLDLR*	0.00150
GOTERM_CC_FAT	GO:0032994	Protein-lipid complex	*APOB, APOA1, CETP, APOVLDLII, VLDLR*	0.00155
GOTERM_CC_FAT	GO:0034358	Plasma lipoprotein particle	*APOB, APOA1, CETP, APOVLDLII, VLDLR*	0.00155
GOTERM_BP_FAT	GO:0010876	Lipid localization	*APOB, TPRXL, APOA1, PPARG, ANXA1, ATP11A, CETP, VTG2, ATP8B3, VLDLR*	0.00167
GOTERM_BP_FAT	GO:0006695	Cholesterol biosynthetic process	*HMGCR, FDPS, LSS, DHCR24*	0.00432
SP_PIR_KEYWORDS		VLDL	*APOB, APOVLDLII, VLDLR*	0.02855
GOTERM_CC_FAT	GO:0034361	Very-low-density lipoprotein particle	*APOB, APOVLDLII, VLDLR*	0.03533
GOTERM_CC_FAT	GO:0034385	Triglyceride-rich lipoprotein particle	*APOB, APOVLDLII, VLDLR*	0.03533
GOTERM_BP_FAT	GO:0006694	Steroid biosynthetic process	*HMGCR, FDPS, LSS, SC4MOL, DHCR24*	0.04625
SP_PIR_KEYWORDS		Lipid transport	*APOB, APOA1, VLDLR*	0.10992

^a^ *GOTERM_BP* GO term under the biological process category, *GOTERM_CC* GO term under the cellular component category, *SP_PIR_KEYWORDS* annotation from the Swiss-Prot and Protein Information Resource databases

To identify critical signal regulation pathways during laying period, we mapped the 960 SDE genes to KEGG orthologs and performed an enrichment analysis with the whole transcriptome as background. The SDE genes were enriched in 13 KEGG pathways and nine of these pathways were significantly (*P* ≤ 0.05) related to steroid biosynthesis, PPAR signaling pathway, biosynthesis of unsaturated fatty acids, glycerophospholipid metabolism, pyruvate metabolism, and four amino acid-related metabolism pathways (Table 4). Additionally, based on the SDE genes pathway enrichment results, the DE genes that were predicted to play important roles in lipid metabolism and involved in PPAR signaling pathway and steroid biosynthesis are shown in the Additional file 6: Figure S1, and Additional file 7: Figure S2.

**Table 4:** KEGG pathways associated with SDE genes between liver samples from juvenile and laying hens

Pathway code	Term	Genes	*P*-value
gga00100	Steroid biosynthesis	*SOAT1, CYP51A1, DHCR7, LSS, HSD17B7, SC4MOL, NSDHL, DHCR24, FDFT1*	5.61E-07
gga03320	PPAR signaling pathway	*SCD, PPARG, FADS2, DBI, APOA1, ACSL1, CYP7A1, FABP3, FABP1, FABP5, ACSL5, ACAA1, ANGPTL4*	7.21E-04
gga00330	Arginine and proline metabolism	*ALDH7A1, ASL2, P4HA2, GATM, ASS1, GLUD1, MAOB, GAMT, AGMAT, PRODH*	0.00128
gga01040	Biosynthesis of unsaturated fatty acids	*PECR, SCD, FADS1, ELOVL2, FADS2, ELOVL6, ACAA1*	0.00144
gga00260	Glycine, serine and threonine metabolism	*GATM, BHMT, MAOB, GCAT, GAMT, PSAT1, GLDC*	0.01192
gga00564	Glycerophospholipid metabolism	*GPD2, GPD1, PLA2G4A, DGKQ, PLA2G12A, GNPAT, ETNK2, PISD, GPAM, AGPAT2*	0.02019
gga00620	Pyruvate metabolism	*ME1, LDHB, ALDH7A1, AKR1B1, PDHA1, ACSS2, PDHX*	0.03314
gga00250	Alanine, aspartate and glutamate metabolism	*ASL2, ASS1, GLUD1, ABAT, GPT2, PPAT*	0.03791
gga00380	Tryptophan metabolism	*KYNU, ALDH7A1, CYP1A1, MAOB, ACMSD, HAAO, AFMID*	0.03816
gga00630	Glyoxylate and dicarboxylate metabolism	*HAO1, PGP, ACO1, AFMID*	0.05155
gga00512	O-Glycan biosynthesis	*GALNT2, GALNT1, GALNT6, WBSCR17, GALNTL1, ST6GALNAC1*	0.05928
gga00280	Valine, leucine and isoleucine degradation	*BCAT1, ALDH6A1, ALDH7A1, BCKDHB, ABAT, HIBADH, ACAA1*	0.06290
gga02010	ABC transporters	*ABCG8, ABCG5, TAP2, ABCB6, ABCA3, ABCG1*	0.09651

## Discussion

Lipid synthesis and transfer is a dynamic and complex process, and previous studies have suggested that the enzymes involved in this process could play different roles in mammal and chicken or other avian species [29]. Indeed, recent studies have shown that some mammalian genes related to lipid metabolism have been lost in chicken [22]. As a consequence, the regulation in gene expression of lipid metabolism in chicken liver is yet to be fully understood.

DE genes are considered to be important regulatory factors of lipid synthesis and transport in liver during the laying stage of chicken. In this study, we obtained a total of 2567 DE genes between juvenile hens and laying hens livers using RNA-Seq technology. Some of these may participate in lipid biological synthesis, assemble, and transfer at the two different physiological stages. For example, *SCD-1* (Stearoyl-CoA desaturase) together with *FADS2* (previously named Δ6 desaturase) were up-regulated in the lipogenesis of the *PPAR* signaling pathway in the liver of laying hen in this study. SCD, which is regulated by a hormone, is a rate limiting enzyme of monounsaturated fatty acid synthesis in liver, and the mRNA expression and activity of *SCD-1* have been shown to be triggered by insulin to promote fat synthesis [51]. *FADS2*, which catalyzes the initial desaturation step to synthesize the long chain polyunsaturated fatty acid (LC-PUFAs), was found to occur mainly in the liver of laying hens [52], suggesting that *FADS2* may contribute to yolk formation. In the liver of severe negative energy balance cows, the down-regulation of *FADS2* was shown to suppress the synthesis of LC-PUFAs, arachidonic acid, and eicosapentaenoic acid [53]. Taken together, these results indicated that the RNA-Seq data generated by this study was sufficiently representative of the chicken liver transcriptome.

VLDL assembly occurs in the endoplasmic reticulum of hepatic cells. To assemble a lipoprotein particle that is competent for transport through the secretory pathway, ApoB has to interact with triglycerides, cholesteryl esters, free cholesterol, and phospholipids. This is a highly regulated process that requires the activity of MTTP [54, 55]. It was reported that chicken MTTP contained functionally important domains that are commonly found in the large lipid transport protein family [56]. However, another study showed that MTTP did not respond to the increase of VLDL induced by estrogen either *in vivo* or *in vitro* [29]. In this study, *MTTP* was not significantly differentially expressed in the liver of laying hens compared to juvenile hens. This study is consistent with a previous finding in the coordinated up-regulation of protein components, such as ApoB (8-fold change) and ApoVLDL-II (320-fold change), along with the up-regulation of lipid synthesis led to increased production of VLDL during an egg-laying cycle in avian [57, 58]. The female-specific yolk precursor proteins VTG1 and VTG2, which are synthesized in the liver and depend on estrogen stimulation [59], were found to be abundantly expressed in the liver of laying hens, and the expression of *VTG2* was the main subtype [60]. Estrogen works via the estrogen receptors (ERα and ERβ) and GPR30 that regulate the transcription of target genes, which contain estrogen response elements. Previous research has suggested that *in vitro*, ERα rather than ERβ and GPR30 could mediate estrogen’s effects on stimulating vitellogenin and ApoVLDL production, while *in vivo*, *ERβ* was up-regulated in liver of laying hen in comparison to  pullet [17]. Our finding is consistent with previous report that the expression of *ERβ* but not *ERα* was significantly up-regulated *in vivo*. Since so many genes are directly or indirectly involved in the complex physiological process in liver during egg-laying stage, it is hard to believe that estrogen mediates these genes only via ERβ. Therefore, these results may still need to be further validated in future studies.

FABPs have been reported to have multiple biological functions, including roles in hepatic fatty acid oxidation [61, 62], intracellular fatty acid transport [63], storage, and export, as well as in cholesterol and phospholipid metabolism [64–66]. In this study, *FABP1* and *FABP3* were both significantly up-regulated in the liver of laying hens compared with juvenile hens, which suggests that they may promote lipid metabolism in the PPAR signaling pathway to meet the requirements of laying eggs. Acyl-CoA binding protein (ACBP, also known as DBI) was reported to act as an endogenous modulator to regulate the levels of gonadal hormones *in vivo* [67]. The transcriptional factor sterol regulatory element binding protein (SREBP-1) and fatty acid synthase (FASN) genes were both found to be elevated coordinately in laying chicken liver that could synthesize fatty acids *de novo* [68], which was consistent with a previous report [69]. Peroxisomal proliferator-activated receptor α (PPARα, a transcriptional factor) controls the expression of fatty acid oxidative metabolism by modulating the expression of peroxisomal acyl-CoA oxidase and mitochondrial carnitine palmitoyltransferase [70], and it has been reported to be highly expressed in rodent liver [71] and swine adipose tissue [72]. However, in the present study, *PPARα* was suppressed in the laying hen liver, suggesting that adipose tissue may oxidize sizeable quantities of fatty acids in avian species, and perhaps also in other mammalian species.

LPGAT1 belongs to a large group of acyltransferases and is a member of the lysophosphatidic acid acyltransferase family. LPGAT1 promotes hepatic lipogenesis in mice [21] and also may be involved in triacylglycerol synthesis and secretion in liver [73]. However, in this study we found that *LPGAT1* was down-regulated in laying hens liver. In addition, in a related study we showed that down-regulated *LPGAT1* was induced by estrogen both *in vivo* and *in vitro* (data not shown). All these results suggested that *LPGAT1* may have different expression patterns in mammals and avian related to specific functions in regulating fatty acid synthesis. Furthermore, *LPGAT1* may have multiple subcellular localizations, and could therefore potentially have multiple functions in different cells or within the same cells [74].

In mammals, lipogenesis is known to occur in liver, adipose tissue, and mammary gland, whereas, in avian species, it occurs mainly in avian liver [71]. During the egg laying stage, fat synthesis in chicken liver is especially active [75]. The GO annotation cluster analyses (Additional file 5: Table S5) showed that the SDE genes were involved mainly in lipid biosynthesis, transport and localization, sterol and cholesterol metabolism, as well as in immune response and some other processes. In poultry, the ovary cannot synthesize lipids; therefore, liver lipoproteins are transferred in the plasma and deposited into the oocytes to form the egg yolk in laying hens. Therefore, lipid synthesis in chicken liver and lipoprotein transfer plays a crucial role on the egg production performance of hens. Some of the SDE genes that are not be involved in lipid metabolism may instead contribute to liver homeostasis in response to the dramatic increase in lipogenesis and protein biosynthesis in the liver of hens at the laying stage.

Alternative splicing of pre-mRNA plays an important role in regulating gene expression in higher eukaryotes. A previous report indicated that 40–60 % of human genes have alternative splicing isoforms, although some variants exist only in relatively low abundance [76]. It has been shown that proteins with different functions can be produced by a diverse array of mRNAs derived from a single pre-mRNA, suggesting that alternative splicing is a crucial mechanism for regulating life [77]. The three alternative splicing isoforms α, δ, and γ of the *PPAR* gene were detected in our transcriptomic data, isoform γ was significantly down-regulated in L30 compared with L20, while isoforms α and δ were not differentially expressed. It has been shown that *PPARα* and *PPARγ* may play significant roles in glucose and lipid metabolism in the early life stage of mouse [78]. Moreover, the DE novel genes detected in this study may provide important information about liver lipid metabolism in chicken. For example, a significantly up-regulated novel gene ENSGALG00000014190 with four alternative splicing isoforms was observed in our transcriptome sequencing data. This gene was predicated to encode a protein of 357 amino acids that could take part in the lipid metabolic process (UniProt: F1NXW6), which requires confirmation. Another up-regulated novel gene ENSGALG00000023444 with three isoforms was also observed, but its isoforms and function need to be investigated further.

Lipid metabolism is controlled by multiple pathways and influenced by multiple genes. These pathways include the PPAR signaling pathway, steroid biosynthesis, steroid hormone biosynthesis, and biosynthesis of unsaturated fatty acids [79]. In our KEGG analysis, the PPAR signaling pathway, which is essential for lipid metabolism, showed one of the most significant associations with the SDE genes in the livers of laying hens. Eighteen DE genes involved in the PPAR pathway (Additional file 6: Figure S1); 11 were up-regulated and seven were down-regulated. In the PPAR pathway, a cytochrome P450 (CYP7A1) catalyzes the rate limiting step of conversion of cholesterol into bile acids. CYP7A1 is also involved in the KEGG Bile secretion pathway, and was reported to be up-regulated in severe negative energy balance cows [53]. The altered expression patterns of hepatic genes in the PPAR signaling pathway could play a role in regulating the lipid metabolism. In addition, a total nine DE genes (Additional file 7: Figure S2) which all were SDE ones were found to be involved in the steroid biosynthesis pathway and all of them were up-regulated except sterol O-acyltransferase (SOAT1, esterification to fatty acids), which suggests that this pathway was quite active in steroid hormone synthesis. SOATs (SOAT1 and SOAT2) are known to synthesize cholesterol fatty acid esters using fatty acids released from membrane phospholipids [80].

During the laying stage, gene expression is highly stimulated in liver to support the metabolic changes associated with the development of the reproductive organs. In the present study, we identified 960 SDE genes with a fold change ≥2 or ≤0.5 (FDR ≤0.05) in the livers of laying hens compared with juvenile hens. Although species-specific differences should be considered when comparing chicken with mammalian systems, the current findings appear to be consistent with conservation of lipid metabolism and adipogenesis processes in chicken and mammal. The chicken liver transcriptome reported here could greatly broaden our understanding of the regulation and networks of gene expression related to liver lipid metabolism in hens at different physiological stages. Our results will serve as important resource for revealing the mechanism of lipid metabolism during egg-laying stage.

## Conclusions

This study generated transcriptomic data using RNA-Seq technology that will help to expand our understanding of the molecular repertoire of lipid metabolism-related genes at different physiological stages in chicken. Differences in expressed genes were found between the juvenile and egg laying stages, including highly expressed novel genes, splice isoforms, and pathways. These findings will be a valuable resource for biological investigations of liver lipid metabolism-related genes in chicken, and may also provide clues for understanding the molecular mechanisms in other poultry and mammalian species.

### Accession numbers

The raw sequencing data, mapped data, and data for visualization of the RNA-Seq analyses of the chicken liver transcriptome data at different physiological stages have been deposited in the Gene Expression Omnibus (GEO) at the National Center for Biotechnology Information (NCBI) under accession number GSE70010.

## Additional files

Additional file 1: Table S1. Primers used for qRT-PCR in this study. (DOCX 15 kb) Additional file 2: Table S2. SDE novel hepatic genes between two different physiological stages (juvenile and egg laying hens). (XLSX 29 kb) Additional file 3: Table S3. SDE hepatic genes between two different physiological stages (juvenile and egg laying hens). (XLSX 110 kb) Additional file 4: Table S4. SDE hepatic isoforms between two different physiological stages (juvenile and egg laying hens). (XLSX 37 kb) Additional file 5: Table S5. Gene ontology annotation clusters of SDE hepatic genes between juvenile and egg laying hens. (XLSX 112 kb) Additional file 6: Figure S1. Changes in hepatic gene expression in the PPAR signaling pathway between juvenile and laying hens. Green boxes indicate down-regulated DE genes detected by RNA-Seq; red boxes indicate up-regulated DE genes. (TIFF 9842 kb) Additional file 7: Figure S2. Changes in hepatic gene expression in the steroid biosynthesis pathway between juvenile and laying hens. Green boxes indicate down-regulated DE genes detected by RNA-Seq; red boxes indicate up-regulated DE genes. (TIFF 9843 kb)
