# Supplementary material for: Transcriptome profile of liver at different physiological stages reveals potential mode for lipid metabolism in laying hens
Source: BMC Genomics. 2015 Oct 9;16:763. doi: 10.1186/s12864-015-1943-0 (PMC4600267; doi:10.1186/s12864-015-1943-0)
Supplement: Additional file 1: Table S1. — Primers used for qRT-PCR in this study. (DOCX 15 kb) [file 12864_2015_1943_MOESM1_ESM.docx]

| Gene | Forward primer | Reverse primer | Production length(bp) |
| --- | --- | --- | --- |
| CEPT1 | TACAGCTTTCATAGGCCCGG | AGAGGACTTGATTCGGAACAC | 184 |
| CETP | CATCGCTGCTGTCTGAATCC | CTCCACACCTTAGCCACAGA | 223 |
| MTP | CAGGAGGGATGGAGTTCAGC | TGGTCACGGAATGCCTGAAA | 243 |
| ApoB | ATGTTCAAAAGATGCGGCCC | GCATGGCTCTTCTCTCACTG | 224 |
| LPGAT1 | GACAACCAACAGTTACACAT | AGGAAAAGCTCCTGTTTCAT | 149 |
| SIRT3 | ATCTTGTAGGACCGTTTGCC | GCCAGCTGTCCTATTTGTCT | 173 |
| FOXO3 | GTCCATCATCCGCAGTGAAC | CAGCAGATTTGGCAAAGGGT | 234 |
| SIRT1 | CAGACCCACGGACAATTCTT | TTTTCTTTTTGGTGGTTCGG | 119 |
| PRDX4 | GACGAGCAGTGCCACTACTA | AGGCGATTATCTCAGTGGGA | 250 |
| RPS24 | GCCACAGTCCCCAAAACAG | TGTTCTTACGTTCCTTCCGC | 241 |
| Novel-gene | TTCATATGAGCACGGTGGGG | GGTCATTCCTGGCTCAAACA | 148 |
| β-actin | GAGAGAAGATGACACAGAC | GTCCATCACAATACCAGTGG | 116 |

**Additional file 1: Table S1** Primers used for qRT-PCR in this study
