# Supplementary figures and images for: Transcriptome profile of liver at different physiological stages reveals potential mode for lipid metabolism in laying hens
Source: BMC Genomics. 2015 Oct 9;16:763. doi: 10.1186/s12864-015-1943-0 (PMC4600267; doi:10.1186/s12864-015-1943-0)

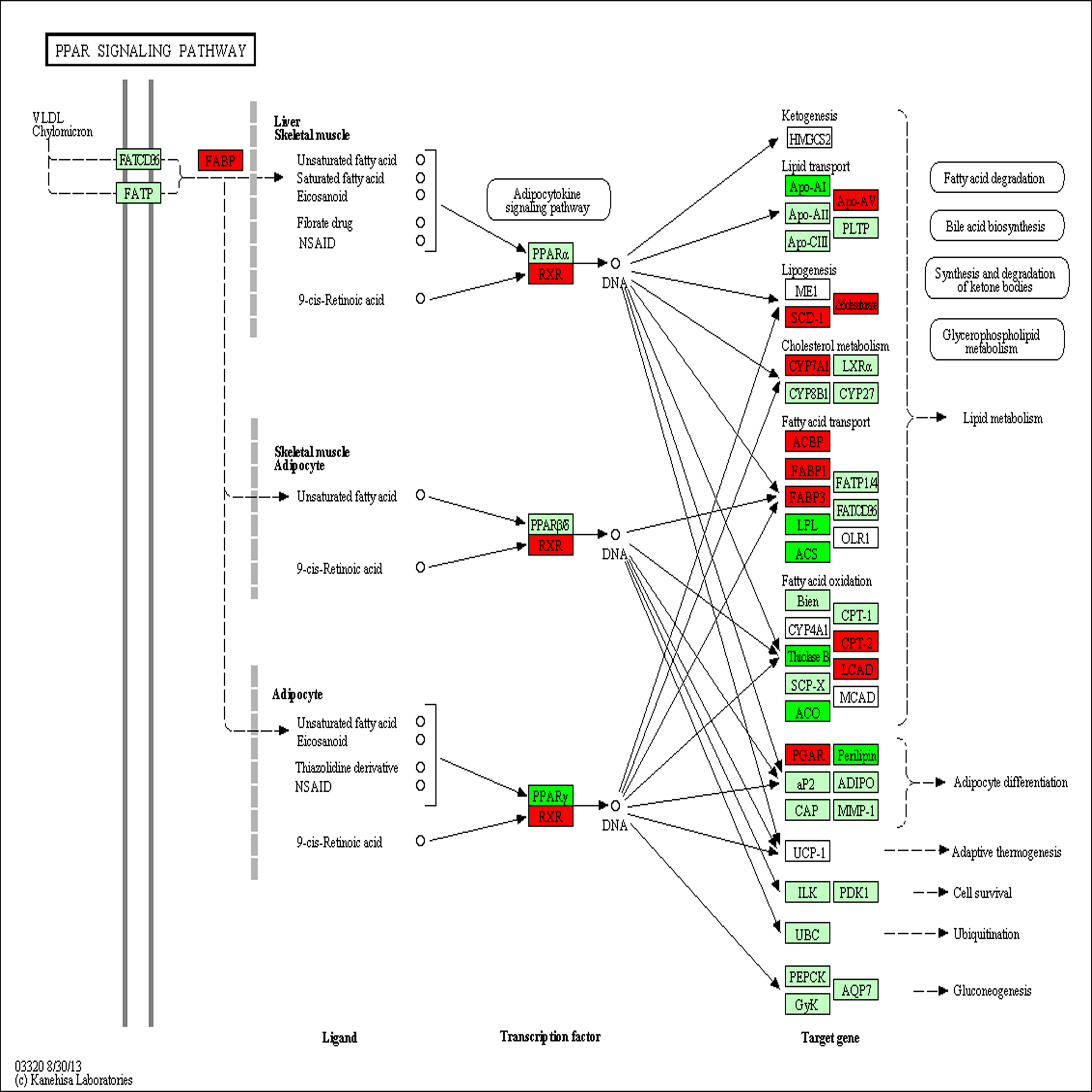

Supplement: Additional file 6: Figure S1. — Changes in hepatic gene expression in the PPAR signaling pathway between juvenile and laying hens. Green boxes indicate down-regulated DE genes detected by RNA-Seq; red boxes indicate up-regulated DE genes. (TIFF 9842 kb) [file 12864_2015_1943_MOESM6_ESM.tif]

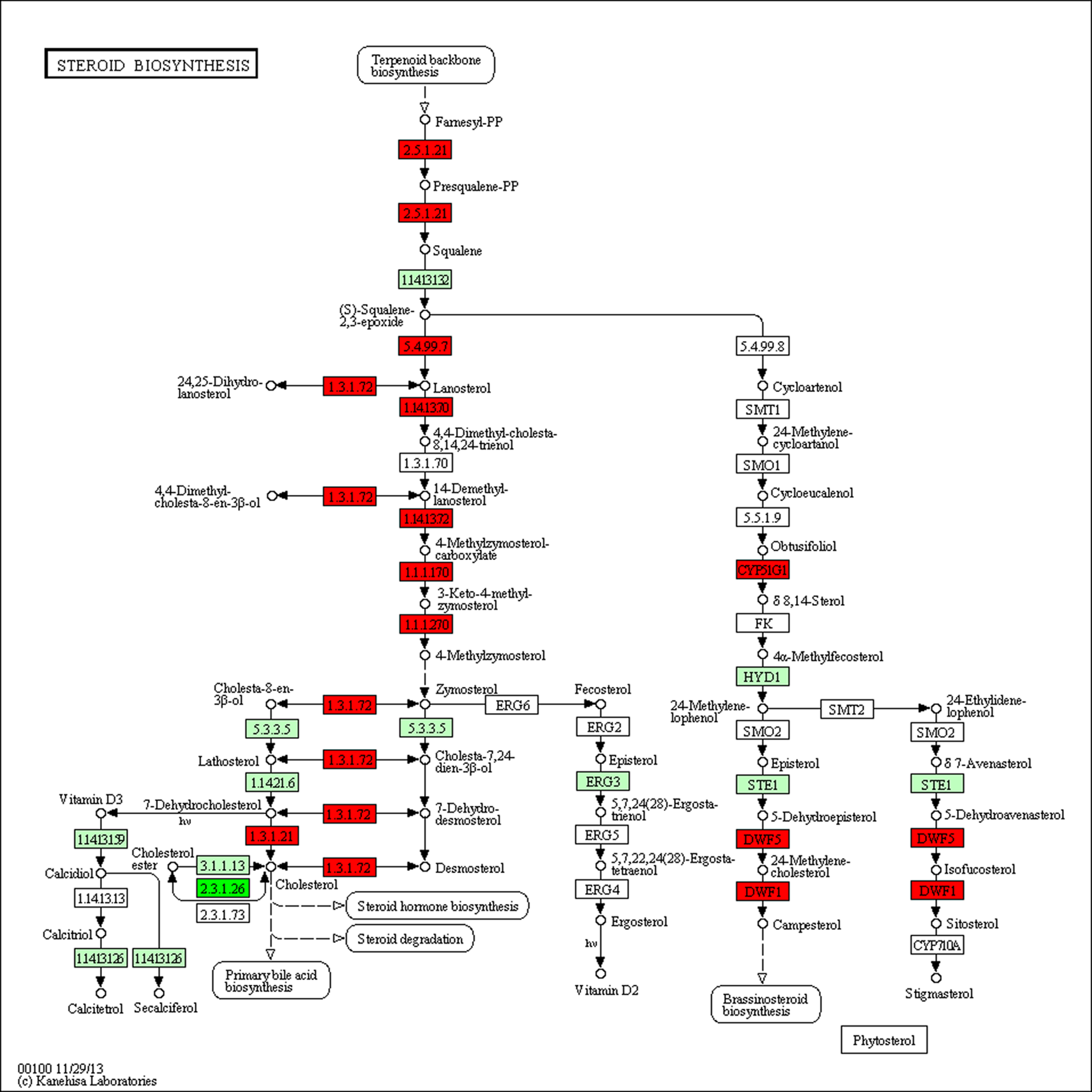

Supplement: Additional file 7: Figure S2. — Changes in hepatic gene expression in the steroid biosynthesis pathway between juvenile and laying hens. Green boxes indicate down-regulated DE genes detected by RNA-Seq; red boxes indicate up-regulated DE genes. (TIFF 9843 kb) [file 12864_2015_1943_MOESM7_ESM.tif]
